# Hydrogen Storage Properties of Ball Milled MgH_2_ with Additives- Ni, V and Activated Carbons Obtained from Different By-Products

**DOI:** 10.3390/ma16206823

**Published:** 2023-10-23

**Authors:** Eli Grigorova, Pavel Markov, Boyko Tsyntsarski, Peter Tzvetkov, Ivanka Stoycheva

**Affiliations:** 1 Institute of General and Inorganic Chemistry, Bulgarian Academy of Sciences, Acad. G. Bonchev Str., Bld. 11, 1113 Sofia, Bulgaria; pvlmarkov@svr.igic.bas.bg (P.M.); ptzvetkov@svr.igic.bas.bg (P.T.); 2 Institute of Organic Chemistry with Centre of Phytochemistry, Bulgarian Academy of Sciences, Acad. G. Bonchev Str., Bld. 9, 1113 Sofia, Bulgaria; boiko_sf@yahoo.com (B.T.); ivanka.stoycheva@orgchm.bas.bg (I.S.)

**Keywords:** sorption, hydrogen storage, metal hydrides, carbon materials

## Abstract

The hydrogen sorption of materials based on 80 wt.% MgH_2_ with the addition of 15 wt.% Ni or V and 5 wt.% activated carbons synthesized from polyolefin wax, a waste product from polyethylene production (POW), walnut shells (CAN), and peach stones (CPS) prepared by milling under an inert Ar atmosphere for a period of 1 h, is investigated. All precursors are submitted to pyrolysis followed by steam activation in order to obtain the activated carbons. The hydrogen sorption evaluations are carried out for absorption at 473 and 573 K with pressure of 1 MPa and for desorption at 623 and 573 K with pressure of 0.15 MPa. The composition of the samples after milling and hydrogenation is monitored by X-ray diffraction analyses. The 80 wt.% MgH_2_–15 wt. %Ni–5 wt.% POW or CAN after absorption–desorption cycling and in a hydrogenated state at 573 K and 1 MPa are analyzed by TEM.

## 1. Introduction

Materials based on solid-state hydrogen storage include adsorbents, liquid organics, complex and interstitial hydrides as well as chemical hydrogen. Each of them has advantages and disadvantages. Some of these materials have higher capacity but poor reversibility, and some have an elevated explosive risk such as, for example, in the case of complex hydrides. Other materials such as ammonia store hydrogen in an irreversible way, and their toxicity and recycling problems are very important. The highly porous adsorbents such as carbon nanotubes and MOFs adsorb hydrogen at a temperature below 100 K and high pressure [1]. Hydrogen storage in the form of metal hydrides provides important safety and high energy density advantages over the gas and liquid storage methods. Among a lot of metals and intermetallics that are capable to react with hydrogen in a reversible way magnesium–based materials as hydrogen storage media are more perspective. There has been intensive research regarding their hydrogen storage application due to the high theoretical hydrogen absorption capacity of magnesium (7.6 wt.%), very good reversibility, abundance, and low cost. These materials have also some fallibilities, such as slow kinetics, necessity of activation, and increased sorption temperatures, especially for the desorption process. Using a variety of dopants combined with milling in a planetary or vibratory mill leads to a decrease in hydrogen sorption temperatures and enhanced kinetics, and in general, to an improvement in the hydrogen absorption–desorption properties. Depending on the nature of the additives, their catalytic effect is different. Some of the additives can easily form hydrides; especially non-stoichiometric and others like iron, for example, act as active sites during the dissociative chemisorption of hydrogen. The amount of additives should be carefully balanced. A larger amount will reduce the hydrogen storage capacity of the material; however, to show a noticeable effect, they must be at least a few percent. When various carbon compounds are milled in mixtures based on Mg or MgH_2_, the hydrogen sorption temperature is reduced, and better kinetics and the impeding of particles agglomeration during milling and hydrogen sorption cycling are observed [2,3,4,5,6,7,8,9,10,11,12]. Ball milling the MgH_2_, which is brittle, is more effective than milling pure magnesium powder. Fuster et al. stated that the catalytic effect of graphite is more pronounced when it has increased content and is introduced at the start of milling. Another aspect of this study showed that the effect of graphite is not connected with its morphology [3]. An amelioration of dehydrogenation process occurs with the increasing carbon content, microwave power, and milling time when MgH_2_ is catalyzed by different carbon materials under microwave irradiation [5]. Various carbon materials added to magnesium in reactive ball milling (milling under hydrogen) resulted in a significant reduction in hydrogenation time compared to pure magnesium [12]. During ball milling, a layer of carbon coating is formed between the particles, and this leads to the significant reduction in the particle size. This carbon layer prevents particles agglomeration and inhibits the restoration of the oxide layer on the surface during repeated hydrogen absorption and desorption. Among the additives used by Rud et al., amorphous carbon and fine-grained graphite powders promote a finer ball milling pulverization of magnesium than other carbon allotropes [12]. The Mg_2_Ni has better sorption kinetics than Mg and absorbed–desorbed hydrogen at moderate temperatures [13,14,15,16]. The ternary hydride of Mg_2_Ni-Mg_2_NiH_4_ crystalizes in the monoclinic and orthorhombic low-temperature phases and high temperature phase with cubic structure. These two low-temperature polymorph phases usually coexist, and their relative ratio depends on the mechanical and thermal history of the alloy and affects the dehydrogenation behavior, electric conductivity, and color of the obtained hydrides. The synthesis of Mg_2_NiH_4_ from MgH_2_ and Ni is reported in [12,14,15], which showed that Mg_2_NiH_4_ has a noticeably higher catalytic efficiency than Ni and Mg_2_Ni. Recently, some results regarding the synergetic effect on the MgH_2_ hydrogen storage properties of two types of additives, with one being the carbon-containing material, have been reported [17,18]. Kajiwara et al. [17] proclaimed that adding carbon nanotubes expanded graphite and single-wall carbon nanotubes to MgH_2_-Nb_2_O_5_ mixtures and resulted in better cycling stability, especially in the case of Mg-Nb_2_O_5_ carbon nanotubes compared to MgH_2_-Nb_2_O_5_. Pawan Soni et al. [18] confirmed the better cycling stability of MgH_2_ when two additives such as carbon spheres and Fe nanoparticles are used. Moreover, their results showed an improvement in the hydrogenation–dehydrogenation properties for MgH_2_-Fe carbon spheres.

The MgH_2_-based hydrogen storage tanks with two types of additives such as NbF_5_ or TiF_4_ and activated carbon or MWCNTs demonstrated very good reversibility by maintaining their hydrogen absorption capacity upon cycling [19,20,21]. Although due to deficient heat management and hydrogen diffusion the sorption performance inside the tank was not homogeneous, these obstacles could be solved by designing and fabricating an appropriate tank equipped with a heat exchanger and gas diffusion pathways.

Some results about the electrochemical activity of high-porosity adsorbents such as hydrogen storage media were published in [22,23,24]. The electrochemical hydrogen storage capacity for the nitrogen-doped graphene foam had an impressive value of 1916.5 mAh/g [22]. Definitely, these types of hydrogen adsorbents have perspectives such as hydrogen storage media.

In view of the above discussion, it seems that the synergetic effect of carbon-containing additives and some metals such as Ni or V could lead to an even greater improvement in the hydrogen absorption–desorption characteristics. We reported already some results about the hydrogen sorption properties of the composites based on MgH_2_ or Mg and dopants as activated carbons from polyolefin wax or apricot stones and Ni, and in some parts, the quantity of the additives were varied [8,9,25]. The MgH_2_-Ni activated carbon and MgH_2_-V activated carbon composites are prepared by milling in a planetary mill under inert atmosphere of argon, and their hydrogen sorption characteristics are tested in this study. The dopants to MgH_2_ are used in the following amounts: 5 wt.% carbon containing one synthesized from organic or agricultural waste precursors and 15 wt.% of Ni or V. Peach stones and walnut shells are widely affordable as precursors in Bulgaria and in the Balkan region and were chosen as plant precursors, and a polyolefin wax is used as organic waste for activated carbon preparation. The tasks of this work are the comparison between the effects of such dopants as Ni or V and the activated carbons from plants and organic wastes in the MgH_2-_based materials, and the synthesis of Mg_2_NiH_4_ in less time, consuming less energy and using more delicate parameters of milling and hydrogenation than those reported to date.

## 2. Materials and Methods

Three different raw materials—polyolefin wax, walnut shells and peach stones—are subjected to pyrolysis first, and after that, they were activated by steam vapor to prepare the porous carbons. They will be denoted further in the text as POW, CAN and CPS. More detailed information about the synthesis procedure of these activated carbons can be found in [8,26]. Dried and crushed peach stones were subjected to pyrolysis and hydro-pyrolysis with water vapor in a tube furnace using a stainless-steel vertical reactor. The solid product was activated by 90 cm^3^/min water vapor. The steam was introduced into the reactor at a temperature of 473 K. The final temperature was 1173 K (1 h, heating rate of 15 K/min). The same synthesis procedure was used for the walnut shells precursor [26]. The polyolefin wax sample is from a Bulgarian petroleum refinery in the city of Burgas, which produces polyethylene under low pressure, and it is situated on the Black Sea coast. Polyolefin wax melts at 388 K, its average molecular weight is 1100 and it decomposes at 633 K. Then, 200 g of polyolefin wax (POW) was heated to a melting point of 388 K. Drops of concentrated sulfuric acid were added while being stirred continuously up to solidification. The resulting solid was cleaned with distilled water before being dried at 423 K and carbonized at 873 K (in a covered silica crucible heated at a rate of 283 K/min filled with nitrogen gas). POW carbonizate was then activated by water vapor for one hour at 1073 K in a vertical stainless-steel reactor. The texture of the synthesized carbon material was characterized by N_2_ adsorption at 77 K, which was carried out in an apparatus Quantachrome Autosorb iQ-C-XR/MP (Anton Paar GmbH, Graz, Austria). Before textural analyses, the sample was outgassed under vacuum at 623 K overnight. The isotherms were used to calculate the specific surface area (SBET), total pore (V_t_) and micropore volumes (V_mic_) by using the Dubinin–Radushkevich method. Analysis of the elements C, H, N and S was carried out with an Elementar Vario MACRO cube apparatus (Elementar Analysensysteme GmbH, Langenselbold, Germany). The oxygen amount in the samples was calculated by the difference.

The MgH_2_ with purity of 98%, Ni powder < 50 µm (99.7%) and V powder 325 mesh (99.5%) are purchased by Sigma Aldrich (St. Louis, MO, USA). The used gases argon (99.999%) and hydrogen (99.999%) are purchased from Messer (Sofia, Bulgaria). Mixtures of MgH_2_, Ni or V and the corresponding carbon additives with the composition of 80 wt.% MgH_2_–15 wt.% Ni or V and 5 wt.% of the activated carbons are ball milled under Ar with applying the following parameters: 200 rpm for a period of 1 h, weight ratio of balls to sample 10:1, stainless steel vial with volume of 80 cm^3^ and balls with 10 mm diameter. The materials were mixed in a glove box by hand, and after that, they were put in a ball milling vessel and closed under argon. For this purpose, a planetary mono mill Pulverisette 6 Fritsch (Fritsch, Idar, Oberstein, Germany) is used. The absorption/desorption characteristics of the composites are studied with a volumetric Sievert-type apparatus. The absorption measurements are accomplished at 473 and 573 K, using a pressure of 1 MPa, while desorption is performed at T = 623 and 573 K and P = 0.15 MPa. The composition after ball milling and hydrogenation is monitored by an XRD powder diffractometer D8 Advance with a LynxEye detector (Bruker, Karlsruhe, Germany) with Cu Kα radiation, vertical θ/θ goniometer, and a step size of 0.02 (2θ). The samples after 10 cycles of hydrogenation/dehydrogenation and in a hydrogenated state at 573 K and 1 MPa are analyzed by TEM HR STEM JEOL JEM 2100 with a CCD Camera GATAN Orius 832 SC1000 (JEOL, Tokyo, Japan) at an accelerating voltage of 200 kV. The specimens are prepared for TEM analyses by dispersing them in acetone. The suspensions are dripped on standard holey carbon/Cu grids purchased from Agar Scientific Ltd., Stansted, Essex, UK and are exposed for a short period of time to air until their transfer into a TEM holder. The samples are kept under argon in a glove box and were exposed to air only shortly for characterization by XRD and TEM.

## 3. Results and Discussion

### 3.1. Synthesis and Characterization of Activated Carbons

In the Table 1, data about the textural parameters of all three synthesized activated carbons are shown. The highest specific surface area has the activated carbon derived from peach stones (CPS), but the other two from polyolefin wax (POW) and walnut shells (CAN) have also a high specific surface area and similar total pore volume. The lowest micropores volume has the activated carbon derived from walnut shells (CAN). The activated carbons from plant precursors contain oxides and carbonates of magnesium and calcium and have increased ash percentage. The only one synthesized from organic waste is POW, and it has very low ash content and the highest oxygen and hydrogen percentage (Table 2). The ash and carbon in POW are 0.1% and 87.4%. The activated carbons differ not only by the pore volume and specific surface area but also by content of ash and carbon and the type of functional groups on the surface. Moreover, the chemical characterization of the activated carbon derived from POW confirms the high content of oxygen surface groups, which characterizes it as a potentially effective adsorbent for gases as well as heavy metals and organics in an aqueous solution [8]. Only the activated carbons derived from plant precursors contain nitrogen.

### 3.2. Hydrogen Sorption Properties

**Figure 1:**
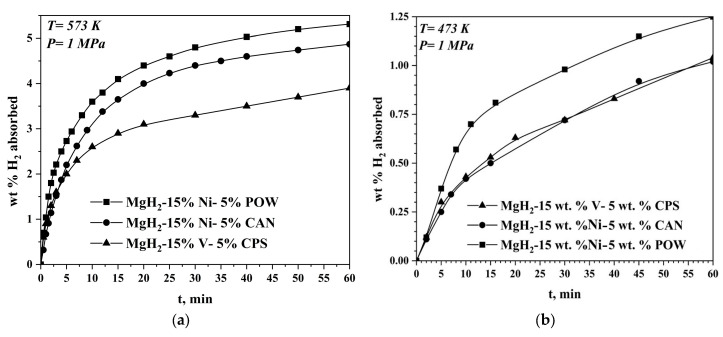
Hydrogen absorption curves of the samples at different temperatures: (**a**) 573 K and (**b**) 473 K and 1 MPa H_2_. The data about the hydrogen absorption curves of the MgH_2_–15 wt.% Ni—5 wt.% POW are published in [25].

The maximal absorption capacity value is 5.3 wt.% H_2_ after 1 h of hydrogenation at 573 K and 1 MPa, and it is the highest one for the sample with Ni and POW additives. For the 80 wt.% MgH_2_–15 wt.% Ni–5wt.% CAN, the absorption capacity is 4.9 wt.% H_2_, and for that with V and CPS, it is 3.8 wt.% H_2_ under the same conditions. The three specimens show a very short activation period. For example, the 80 wt.% MgH_2_–15 wt.% Ni–5wt.% CAN reaches in its first hydrogenation cycle at 573 K and 1 MPa a hydrogen absorption capacity of 0.7 wt.%, while in the second cycle, it reaches 2.4 wt.%, and already in the third one, the kinetics is improved significantly and 4.5 wt.% is obtained. The other two samples’ behavior is similar, and they also need a few cycles of activation. This shows that the complex hydride Mg_2_NiH_4_ is relatively easy to be formed, and it is present in the samples only after several cycles of hydrogenation and dehydrogenation. Reducing the temperature of absorption to 473 K results in a substantial deterioration of the kinetics and decrease in absorption capacity (Figure 1a,b). The rate of absorption is very fast, and all three materials absorb for 5 min. from 2 wt.% to up to 2.73 wt.% of hydrogen. The composite containing vanadium is tested for hydrogen absorption for longer period of 2 h at 573 K and 1 MPa, and its hydrogen absorption capacity after that is 4.3 wt.%H_2_. At 473 K and 1 MPa after 1 h of hydrogen absorption, the three composites reached absorption capacity as follows: 1.25 wt.% for the 80 wt.% MgH_2_–15 wt.% Ni–5wt.% POW and 1.04 wt.% for the 80 wt.% MgH_2_–15 wt.% Ni–5wt.% CAN and for the 80 wt.% MgH_2_–15 wt.% V–5 wt.% CPS. The Mg_2_NiH_4_ is less stable than MgH_2_, e.g., the equilibrium pressure plateau for this ternary hydride is higher. The transformation of the cubic high-temperature phase of Mg_2_NiH_4_ into the low-temperature forms occurs at 508 K. This hydride acts like a “hydrogen pump”, accelerating the hydrogen absorption and desorption of Mg. The capacity of the desorption process at 623 K and 0.15 MPa has a similar value for both samples with Ni, but the reaction rate is higher for the one containing POW. As in the absorption process, the desorption process at a lower temperature of 573 K is faster, and a higher capacity is reached for the POW and nickel-containing sample. Up to 10 min from the start of the desorption reaction at 573 K, its rate is comparable with that at 623 K for the same sample with POW and Ni.

**Figure 2:**
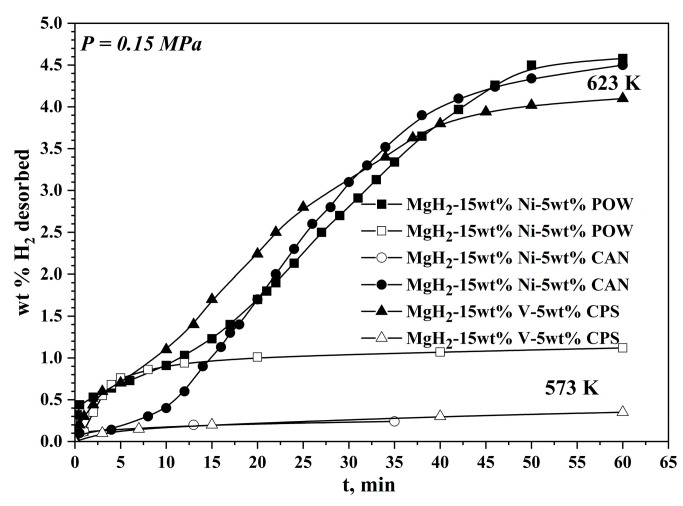
Hydrogen desorption curves of the samples at different temperatures and 0.15 MPa H_2_. The data about the hydrogen desorption curves of the MgH_2_–15 wt.% Ni–5 wt.% POW are published in [25].

The above-mentioned fact that Mg_2_NiH_4_ is less stable than MgH_2_ and desorbs hydrogen at the beginning of the reaction is clearly visible in Figure 2. The desorption curves of both samples with Ni have rates up to 5 min faster than the one with V. Decreasing the temperature of desorption with only 323 K leads to a significant deterioration of the hydrogen desorption properties of studied materials, and after 1 h of desorption process, only about 20% of absorbed hydrogen is desorbed, and for the composites with activated carbons from plant precursors, this percentage is even lower. At the lower temperature of 533 K, the desorption was not detected for any of the three specimens.

The formation of the Mg_2_NiH_4_ hydride has a bidirectional effect. On the one hand, this hydride affects favorably the kinetics of hydrogenation and dehydrogenation, but on the other hand, Mg_2_NiH_4_ reduces the obtained capacity, because the theoretical hydrogen storage capacity of the Mg_2_Ni is more than twofold lower than that of Mg, i.e., 3.6 wt.% and 7.6 wt.%.

The comparison of our results with some already published shows similar hydrogen sorption capacity of about 5.33 wt.% H_2_ after cycling for the MgH_2_–Nb_2_O_5_–carbon nanotube but a better rate of the reactions [17]. It should be pointed out that the absorption is tested at 1.9 MPa; the preparation methods, the composition and one of the additives are different. For the MgH_2_ with 5 wt.% NiO/Al_2_O_3_ layered hybrid, the obtained absorption capacity is similar, e.g., around 5 wt.%, but at 523 K and 3 MPa. Another composite prepared and studied in this paper is MgH_2_ with a 5 wt.% Ni/Al_2_O_3_ layered hybrid, which absorbed close to 6 wt.% H_2_ at the same temperature and pressure [27]. Definitely, the higher pressure of absorption and the elevated percentage of MgH_2_ are the reason for the better sorption properties of the MgH_2_ with 5 wt.% Ni/Al_2_O_3_ layered hybrid than those reported in this study.

### 3.3. Characterization—XRD and TEM Analyses

X-ray diffraction patterns of the ball milled under argon for a period of 1 h with a composition of 80 wt.% MgH_2_–15 wt.% Ni–5 wt.% POW, 80 wt.% MgH_2_–15 wt.%–Ni-5 wt.%–CAN and 80 wt.% MgH_2_–15 wt.% V–5 wt.% CPS mixtures do not show appearance of new phases, but regarding the relatively short milling time, it is not unexpected. The detected phases in the ball milled composites are mainly tetragonal MgH_2_ and also Ni or V and some Mg (not presented). These grinding conditions were chosen because of the goal of the ball milling, which was mostly used for good homogenization of the studied materials.

**Figure 3:**
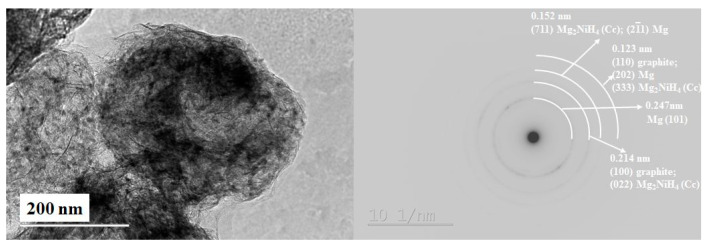
TEM image (**left**) and polycrystalline electron diffraction (**right**) of the 80 wt.% MgH_2_–15 wt.% Ni–5 wt.% POW hydrogenation and cycling at 573 K and 1 MPa H_2_ [25].

**Figure 4:**
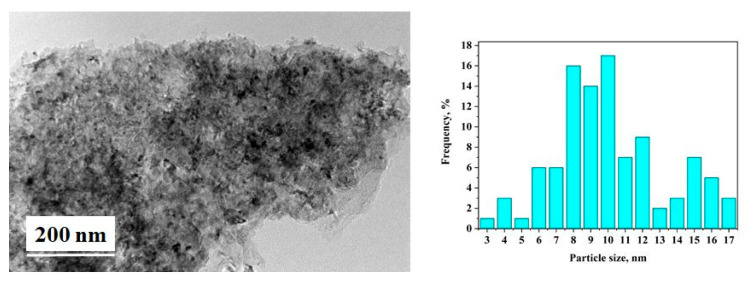
TEM image (**left**) and particles size distribution (**right**) of the 80 wt.% MgH_2_–15 wt.% Ni–5 wt.% CAN after hydrogenation and cycling at 573 K and 1 MPa H_2_.

**Figure 5:**
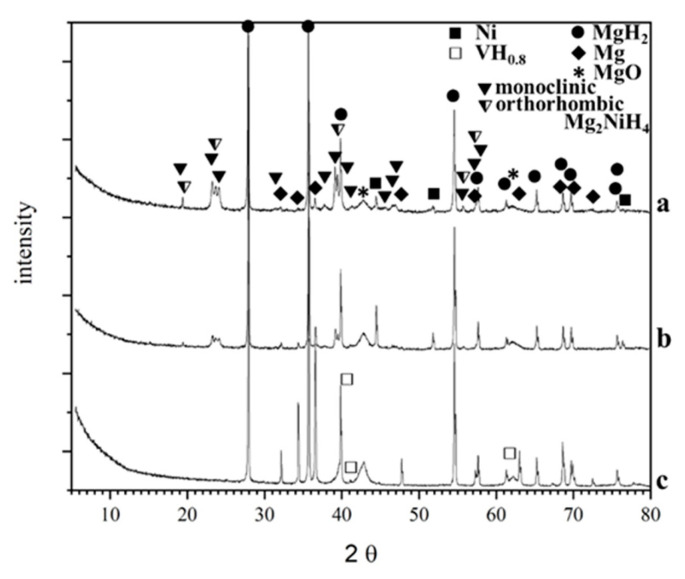
X-ray diffraction patterns obtained after hydrogenation and cycling at T = 573 K and P = 1 MPa of the: (a) MgH_2_–15 wt.% Ni–5 wt.% POW; (b) MgH_2_–15 wt.% Ni–5 wt.% CAN and (c) MgH_2_–15 wt.% V–5 wt.% CPS.

The characterization of these types of materials by TEM is somehow challenging because of their oxygen and moisture sensitivity and the elevated energy of the electron beam, which can negatively affect them. The characterization by TEM of these sensitive materials, particularly high-resolution transmission electron microscopy (HRTEM) and selected area electron diffraction (SAED), is obtained by low beam current values and as short as possible exposition times. The XRD and the TEM analyses, e.g., SAED and HRTEM at different areas of the samples after hydrogenation and ten cycles of hydrogen absorption and desorption, confirmed the presence of MgH_2_ as the main phase and Mg_2_NiH_4_ with a monoclinic and orthorhombic structure, which are the two polymorphic configurations of a low-temperature phase along with some graphite, Mg, Ni and MgO (Figure 3, Figure 4 and Figure 5).

For the composite 80 wt.% MgH_2_–15 wt.% V–5 wt.% CPS, instead of ternary hydride Mg_2_NiH_4_, the non-stoichiometric hydride of vanadium VH_x_ is detected after hydrogenation and cycling (Figure 5). Using the Image J program for image analysis, the particle size distribution is obtained for the 80 wt.% MgH_2_–15 wt.% Ni–5 wt.% POW or CAN, and the average particles size diameter is 8 nm for that containing POW and 9 nm for that with CAN (Figure 4). The particles size for both composites is between a few nm and less than 20 nm. After hydrogenation and cycling, the composites are more sensitive to oxidation, and the presence of MgO is very clearly visible on the XRD patterns. Another observation is that for the 80 wt.% MgH_2_–15 wt.% V–5 wt.% CPS, there is much more nonhydrogenated magnesium compared to the other two composites after 1 h of hydrogenation at a temperature 573 K and pressure of 1MPa (Figure 5).

The comparison between the composite based on MgH_2_ and only with activated carbons addition with these in the current study shows that the doping with nickel or vanadium as well as a carbon-containing additive leads to some desorption at a lower temperature of 573 K, as well as a comparable absorption rate, but it also leads to a lower capacity value. It should be noted that MgH_2_ composites containing only activated carbons as an additive have a higher theoretical capacity, and the 95 wt.% MgH_2_–5 wt.% AS (activated carbon derived from apricot stones) reached 5.8 wt.%, and for the 95 wt.% MgH_2_–5 wt.% POW, the capacity value was 5.4 wt.% at 573 K and 1 MPa. Despite the higher absorption capacity of the composites only with the addition of activated carbons to MgH_2_, their kinetics of absorption/desorption is slower and the desorption process was detectable only at 623 K [9]. Using plants and other waste precursors for obtaining activated carbons with high surface and developed pore structure is a very important topic in a view of ecology and more specifically in a waste recovery. The obtained materials are nanosized and showed relatively high absorption capacity values, especially this one with Ni and POW additives.

## 4. Conclusions

The maximal obtained absorption capacity value is 5.3 wt.% H_2_ for the sample with the addition of POW and Ni, 4.9 wt.% H_2_ for that with CAN and Ni, and 3.8 wt.% for the last one with V and CPS at a temperature of 573 K and a pressure of 1 MPa after 1 h of hydrogenation. Only after 3 min. of hydrogenation at 573 K, the MgH_2_-based composite with Ni and POW absorbs 2.2 wt.% H_2_. The hydrogen desorption capacities at a temperature of 623 K and a pressure of 0.15 MPa are with close values for the samples with Ni and with an increased rate of desorption for that containing POW. The presence of Ni in two of the studied composites leads to the formation of ternary hydride of Mg_2_Ni and a low temperature form of Mg_2_NiH_4_ with monoclinic and orthorhombic structures, which is more favorable to the sorption kinetics and the absorption capacity than the vanadium addition and formation of its nonstoichiometric hydride. The combination between these two types of dopants with different natures leads to the conclusion that a more pronounced positive effect is activated carbon from organic waste with a high specific surface area, a more developed pore structure, lower ash content and a higher presence of oxygen containing surface groups and Ni because of the Mg_2_NiH_4_ formation. The activated carbons produced from biomass or plant wastes have some advantages such as eco-friendliness and low coast. Our results also showed that a more pronounced impact on the hydrogen storage properties of magnesium-based materials is that there are activated carbons derived from organic wastes. Moreover, the metal type of additive has a superior effect on the absorption–desorption characteristics of magnesium than the type of activated carbon.

## Figures and Tables

**Table 1:** Textural parameters of the activated carbons.

Activated Carbons	Specific Surface Area, m^2^/g	V_tot_, cm^3^/g	V_mic_, cm^3^/g
Walnut shells (CAN)	743	0.67	0.21
Polyolefin wax (POW)	800	0.60	0.27
Peach stones (CPS) [26]	1257	0.63	0.44

**Table 2:** Analyses of the elements.

Type of Activated Carbon	Ash, wt.%	C, wt.%	H, wt.%	S, wt.%	N, wt.%	O, wt.%
Walnut shells (CAN)	2	89.5	2.4	0.8	0.9	6.4
Polyolefin wax (POW)	0.1	87.4	3.5	0.5	-	8.6
Peach stones (CPS) [26]	2.6	88.0	2.5	0.5	1.1	7.9

## Data Availability

The data presented in this study are available on request from the corresponding author.
